# Effectiveness and safety of electroacupuncture combined with conventional drugs in the treatment of stable angina pectoris in coronary artery disease: A systematic evaluation and meta-analysis

**DOI:** 10.1097/MD.0000000000032960

**Published:** 2023-02-17

**Authors:** Shuwen Pang, Yajuan Lv, Wenfang Zhong, Jing Qian, Yueli Zhao, Jiahui Zhong, Weiyi Huang

**Affiliations:** a General Hospital of the Southern War Zone of the Chinese People’s Liberation Army, Guangzhou, China; b Graduate School of Guangzhou University of Chinese Medicine, Guangzhou, China; c Guangdong Hydropower Hospital, Guangzhou, China; d Guangdong Provincial People’s Hospital, Guangzhou, China.

**Keywords:** conventional drugs, coronary artery disease, electroacupuncture, meta-analysis, stable angina pectoris

## Abstract

The objective of this study is to systematically evaluate the clinical effectiveness and safety of electroacupuncture combined with conventional drugs in the treatment of stable angina pectoris.

**Methods:**

Computer searches of 3 Chinese literature databases (CNKI, VIP, WangFang) and 4 English literature databases (PubMed, Embase, Cochrane Central Register of Controlled Trials, Web of Science), all searched from the time of database construction to October 2022. Two researchers were selected to independently perform literature screening, data extraction, and risk of bias evaluation, and meta-analysis of the included studies was performed using RevMan 5.3 software.

**Results:**

A total of 7 publications with a total of 1042 patients were included, and electroacupuncture combined with conventional drug therapy compared with drug therapy alone was effective in improving clinical symptoms of angina pectoris (relative risk [RR] = 1.19, 95% CI = [1.09, 1.31], *P* = .0002), clinical treatment efficiency of electrocardiography (RR = 1.34, 95% CI = [1.19, 1.50], *P* = .00001), visual analog score (VAS) (mean deviation = 0.07, 95% CI = [−0.11, 0.25], *P* = .44), and Seattle Angina Scale (mean deviation = 4.91, 95% CI = [2.91, 6.91], *P* < .00001) were better than conventional drug therapy, while the number of adverse events in the intervention group was lower than that in the control group. One of the outcome indicators with greater heterogeneity was tested by sensitivity analysis, and each outcome indicator was found to be more robust. The risk of bias evaluation of each outcome indicator using funnel plots suggested the possibility of publication bias.

**Conclusion:**

The current study results found that electroacupuncture combined with conventional drugs can significantly improve the clinical symptoms of patients with stable angina pectoris compared with conventional drug therapy, with a low incidence of adverse reactions, but the number of high-quality literature with rigorous study design protocols is currently low, which may cause bias in the results of this study, so the above conclusions need to be further verified through clinical trials.

## 1. Introduction

Stable angina is a clinical syndrome in which patients with coronary artery disease experience acute, temporary ischemia or hypoxia of myocardial tissue due to physical exertion, emotional stimuli, and other external triggers, resulting in chest pain or chest discomfort.^[1]^ According to epidemiological findings, cardiovascular disease deaths account for the first cause of total deaths worldwide, of which 80% of cardiovascular disease deaths are caused by coronary heart disease (CHD), resulting in about 9.14 million deaths worldwide.^[2]^ According to the latest epidemiological studies, CHD is now the leading cause of death among the global population and has become one of the most urgent public health problems.^[3]^ At present, modern medical treatment of CHD mainly adopts the methods of controlling risk factors, standardizing drug intake, intervening in lifestyle and rebuilding blood flow, which can achieve certain efficacy. However, the economic burden, decreased drug tolerance, and drug side effects arising from long-term drug therapy are limitations, so it is necessary to seek natural, safe and effective complementary alternative therapies for the treatment of stable angina.^[4]^

Acupuncture is an effective and safe traditional nonpharmacological treatment for cardiovascular diseases, which has been widely used in the prevention and treatment of CHD because of its clinical efficacy and low adverse effects. Clinical studies have shown that electroacupuncture combined with acupuncture has good efficacy in the treatment of angina pectoris in CHD, which can significantly reduce the symptoms of angina pectoris, improve myocardial ischemia, and promote the recovery of cardiac function. In addition, the study on the efficacy mechanism of electroacupuncture found that electroacupuncture can inhibit the production of inflammatory factors in rats, and reduce chronic pain and other complications while effectively promoting the recovery of neurological function. The use of acupuncture in the early stage of the disease can improve the therapeutic effect, shorten the course of the disease, control the progress of the disease, and improve the quality of life of patients^.^ In addition, electroacupuncture has the unique advantages of being economical, safe, and without significant side effects, which can reduce the economic burden of patients and is worthy of clinical promotion and application. However, the quality of existing studies also varies, and there is a lack of a more complete systematic evaluation of evidence-based medicine. Therefore, this study systematically evaluates the clinical effectiveness and safety of electroacupuncture for stable angina pectoris in coronary artery disease and provides a reference basis and reference for clinical and research purposes.

## 2. Materials and methods

### 2.1. Registration of selected topics

This system evaluation is registered on PROSPERO (registration number CRD42022364351) and was analyzed according to the Preferred Reporting Items for System Evaluation and Meta-Analysis (PRISMA) guidelines.

### 2.2. Search strategy

Pudmed, cochrane library, Embase, Web of Science, CNKI, WangFang, VIP, and CBM were searched by computer to find information about electroacupuncture combined with conventional drugs for the treatment of The literature related to randomized controlled trials on the treatment of stable angina pectoris in coronary artery disease. The literature was searched from the date of database creation to October 6, 2022. All searches were performed using a combination of subject terms and free words, and were supplemented by manual searches for relevant systematic evaluations. The Chinese search terms were “electroacupuncture,” “electroacupuncture therapy,” “coronary atherosclerotic heart disease stable angina,” “coronary heart disease stable angina,” and “coronary heart disease stable angina,” “stable angina,” “angina pectoris,” “randomized controlled trial,” and “randomized controlled trial.” The English search terms consisted of subject terms and free words, “Stable angina,” “Anginas, Stable,” “Chronic Stable Angina “, “Stable Angina Pectori,” “Stable Angina Pectoris,” “randomized controlled trial,” “controlled clinical trial,” “randomized,” “placebo.” Taking PudMed as an example, the search strategy is shown in Table 1.

**Table 1:** PubMed search strategy.

Steps	Strategy
#1	“angina, stable”[MeSH Terms]
#2	(Anginas, Stable[Title/Abstract]) OR (Stable Angina[Title/Abstract])) OR (Stable Anginas[Title/Abstract])) OR (Chronic Stable Angina[Title/Abstract])) OR (Angina, Chronic Stable[Title/Abstract])) OR (Anginas, Chronic Stable[Title/Abstract])) OR (Chronic Stable Anginas[Title/Abstract])) OR (Stable Angina, Chronic[Title/Abstract])) OR (Stable Anginas, Chronic[Title/Abstract])) OR (Angina Pectoris, Stable[Title/Abstract])) OR (Angina Pectori, Stable[Title/Abstract])) OR (Pectori, Stable Angina[Title/Abstract])) OR (Pectoris, Stable Angina[Title/Abstract])) OR (Stable Angina Pectori[Title/Abstract])) OR (Stable Angina Pectoris[Title/Abstract])
#3	(“Angina, Stable”[Mesh]) OR (“Angina, Stable”[Mesh])
#4	“Electroacupuncture”[Mesh]
#5	Electroacupuncture[Title/Abstract]
#6	(“Electroacupuncture”[Mesh]) OR (Electroacupuncture[Title/Abstract])
#7	“Randomized Controlled Trial” [Publication Type]
#8	(((controlled clinical trial[Title/Abstract]) OR (randomized[Title/Abstract])) OR (placebo[Title/Abstract])) OR (RCT[Title/Abstract])
#9	(“Randomized Controlled Trial” [Publication Type]) OR ((((controlled clinical trial[Title/Abstract]) OR (randomized[Title/Abstract])) OR (placebo[Title/Abstract])) OR (RCT[Title/Abstract]))
#10	(((“Angina, Stable”[Mesh]) OR (“Angina, Stable”[Mesh])) AND ((“Electroacupuncture”[Mesh])OR(Electroacupuncture[Title/Abstract]))) AND ((“Randomized Controlled Trial” [PublicationType])OR((((controlledclinicaltrial[Title/Abstract])OR(randomized[Title/Abstract])) OR (placebo[Title/Abstract])) OR (RCT[Title/Abstract])))

### 2.3. Inclusion and exclusion criteria

#### 2.3.1. Inclusion criteria.

Type of study: all published randomized controlled trials on electroacupuncture combined with conventional drugs for stable angina pectoris, without limiting the randomized protocol, without limiting whether blinding was used in the study, and without limiting the language. Study subjects: Western medicine diagnosis was in accordance with the Nomenclature and Diagnostic Criteria for Ischemic Heart Disease promulgated by the International Society of Cardiology and the World Health Organization in 1979.^[5]^ Chinese medicine diagnosis and evidence-based typing conformed to the Chinese Diagnostic and Efficacy Criteria for Diseases and the Guidelines for Clinical Research on New Chinese Medicines, and there were no obvious complications or contraindications to electroacupuncture treatment. Age, gender and race of patients with stable angina pectoris of CHD were not restricted.^[6]^ Interventions: patients in the intervention group were treated with conventional western drugs (nitrates, aspirin, β-blockers, angiotensin-converting enzyme inhibitors and other drugs, ensuring that both groups remained consistent when conventional treatment was involved). Combined electroacupuncture treatment (electroacupuncture machine was used for electrical stimulation after the milli-needle was pierced into the acupuncture point; the selection of acupuncture points, the implementation of the technique, the retention time, the duration of treatment, and the specification and manufacturer of the selected needle and electroacupuncture instrument were not limited). Control measures: patients in the control group were given conventional Western medicine or conventional Western medicine plus simple acupuncture treatment. Outcome indicators: effectiveness indicators: efficiency of improvement of angina symptoms, efficiency of improvement of electrocardiogram, Seattle Angina Scale, visual analog score (VAS) score; safety indicators: incidence of adverse reactions that occurred in patients during treatment.

#### 2.3.2. Exclusion criteria.

Exclusion of nonrandomized controlled trials; clinical studies that did not meet the diagnostic criteria of Chinese and Western medicine for CHD or had incorrect diagnostic criteria; clinical randomized controlled trials in which electroacupuncture was not adopted as the main intervention; literature of review, descriptive, retrospective or personal experience type; literature of in vitro, animal and other nonclinical trial types; literature with duplicate data or multiple submissions of 1 manuscript literature; literature that does not provide outcome indicators for analysis or incomplete data.

### 2.4. Literature screening and data extraction

#### 2.4.1. Literature screening.

The literature retrieved from each database was imported into the literature management software EndnoteX9 (Version X9, https://endnote.com/) to eliminate duplicates, and then 2 systematically trained researchers manually eliminated the literature that did not meet the criteria by reading the subject, abstract and content of the literature according to the inclusion and exclusion criteria, and the 2 researchers cross-checked the screened content, and when they encountered disagreement and could not reach agreement, a third-party gave an opinion.

#### 2.4.2. Data extraction.

Extraction of important information and data in the literature meeting the criteria: basic information of the literature: time of publication, name of the first author, source of the literature, number of subjects; basic information of the study subjects: study inclusion sample size, age of the study subjects; interventions: interventions and duration of treatment and control groups; outcome indicators: efficiency of angina symptom improvement, efficiency of electrocardiogram (ECG) improvement, Seattle Angina Scale, VAS score, incidence of adverse effects; key elements of risk of bias evaluation: randomization method, allocation of hidden protocols, blinding method, etc.

### 2.5. Risk of bias evaluation

Risk of bias evaluation of the literature was performed using the assessment tool recommended by the Cochrane Centre for Evidence-Based Assessment for: randomization scheme, implementation of allocation concealment scheme, implementation of blinding, completeness of outcome data, selective reporting bias, other sources of bias. The literature was evaluated by 2 researchers independently, with further discussion or third-party assessment in case of disagreement.

### 2.6. Statistical analysis

In this paper, Rev Man 5.3 software (Version 5.3, http://ims.cochrane.org/revman/download) was applied to analyze the data from the literature, and the data were statistically analyzed using Relative Risk (RR) and Mean Deviation for counting units and measurement units, respectively, where the effect models were chosen: Fixed-effect model: when the consistency of the study results was high (*P* > .10), both effect sizes were expressed by 95% CI, and when *P* > .05, the difference was not statistically significant; when *P* < .05, the difference was statistically significant. Random-effects model: This model was used when there was heterogeneity in the study, but the differences were not clinically significant. The reasons for the existence of heterogeneity were also analyzed and described, and subgroup analysis was performed when necessary.

## 3. Results

### 3.1. Literature search process

**Figure 1.**
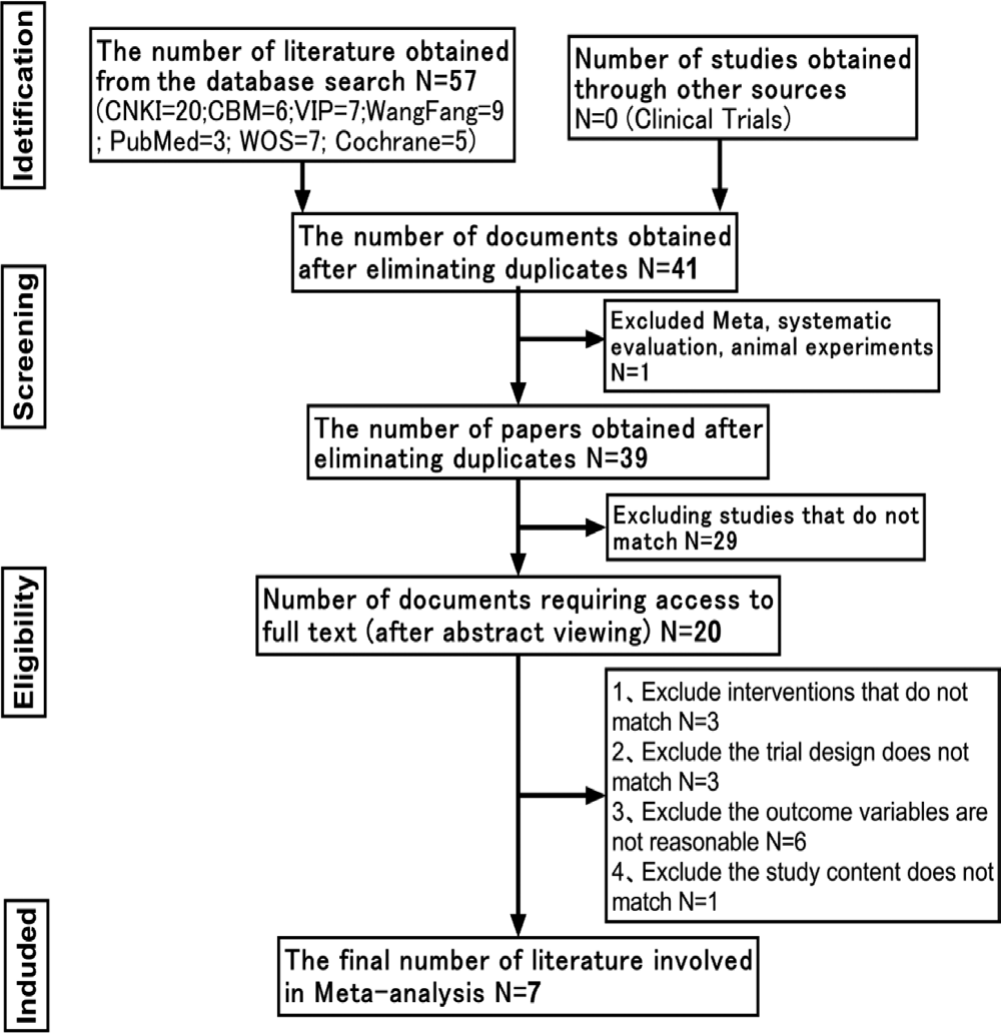
Flow chart for inclusion in the literature.

The initial search yielded 57 documents, and after repeated screening, 7^[7–13]^ documents were finally included (Fig. 1).

### 3.2. Basic characteristics of the included literature

**Figure 2.**
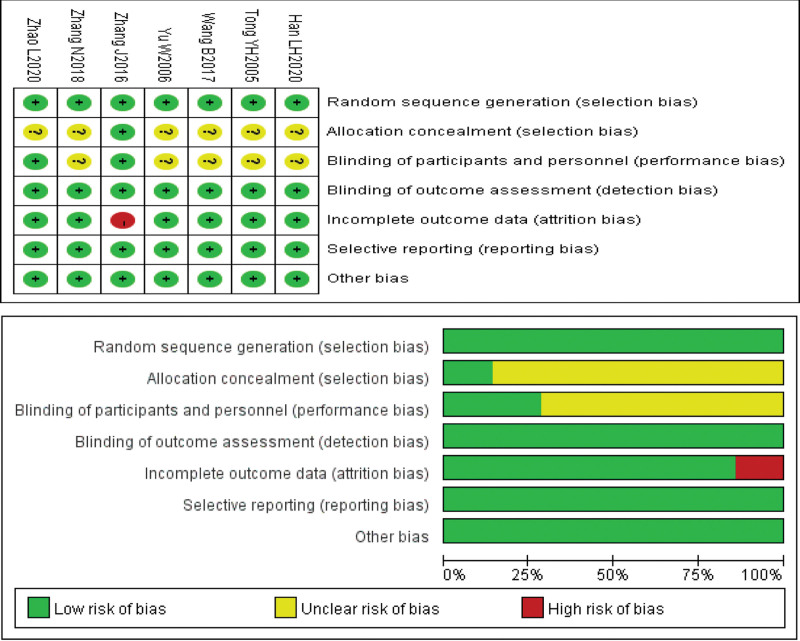
Risk of bias evaluation results.

Six of the included literature were randomized controlled trials, with baseline information for the control and intervention groups described in the text and all comparable, with 6 studies describing specific randomization methods, 2 studies mentioning blinding of subjects, 1 study mentioning shedding data, 1 study mentioning allocation concealment, and Cochrane Collaboration Network risk of bias assessment of the literature (Tables 2 and 3 and Fig. 2).

**Table 2:** Basic characteristics of the included studies.

Studies	Publication time	Sample size		Age		Interventions		Treatment	Closing indicators
		T	C	T	C	T	C		
Tong YH^[11]^	2005	120	80	62.5 ± 8.7	63.4 ± 8.2	Electroacupuncture + Western medicine	Western medicine	10	①, ②
Yu W^[20]^	2006	30	30	67.30 ± 6.67	65.74 ± 6.35	Electroacupuncture + Western medicine	Western medicine	10	①, ②
Zhang J^[21]^	2016	92	94	62.09 ± 9.62	63.35 ± 10.33	Electroacupuncture + Western medicine	Western medicine	28	①, ③
Wang B^[22]^	2017	45	45	61.51 ± 4.95	62.11 ± 5.42	Electroacupuncture + Western medicine	Western medicine	10	①, ②, ⑤
Zhang N^[23]^	2018	30	30	61.12 ± 7.45	60.12 ± 4.45	Electroacupuncture + Western medicine	Western medicine	28	④, ②
Zhao L^[24]^	2020	30	30	60.23 ± 7.09	61.90 ± 8.67	Electroacupuncture + Western medicine	Western medicine	14	③
Han LH^[25]^	2020	102	102	57.54 ± 3.19	56.97 ± 3.77	Electroacupuncture + Western medicine	Western medicine	15	⑤

① Clinical symptoms improvement rate of angina pectoris; ② ECG efficacy efficiency rate; ③ Incidence of adverse effects; ④ VAS score; ⑤ SQA score.

SQA = Seattle Angina Scale score, VAS = visual analog score.

**Table 3:** Inclusion of study risk bias evaluation.

Publication time	Author	Random assignment method	Distribution program hidden	Blind method	Integrity of result data	Selective reporting of study results	Other sources of bias
NO.1	Yu W	Completely randomized grouping	Unclear	Unclear	Unclear	Unclear	Unclear
NO.2	Zhang J	Central random grouping	Hidden	Triple blind	Shedding 18 cases	Unclear	Unclear
NO.3	Zhang N	Random number grouping	Unclear	Unclear	Unclear	Unclear	Unclear
NO.4	Zhao L	Completely randomized grouping	hidden	Single-blind	Unclear	Unclear	Unclear
NO.5	Wang B	Random grouping	Unclear	Unclear	Unclear	Unclear	Unclear
NO.6	Han LH	Random number table method	Unclear	Unclear	Unclear	Unclear	Unclear
NO.7	Tong YH	Unclear	Unclear	Unclear	Unclear	Unclear	Unclear

### 3.3. Meta-analysis results

#### 3.3.1. Efficacy in improving clinical symptoms of angina pectoris.

**Figure 3.**
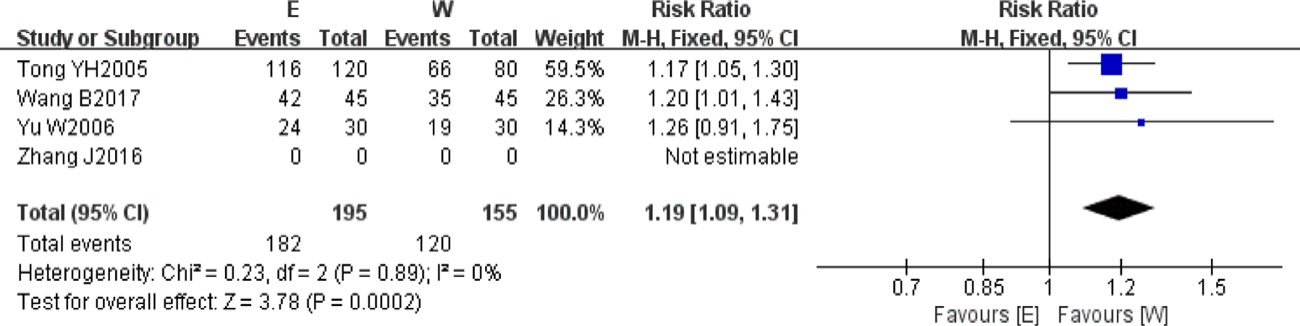
Comparison of the efficacy of angina pectoris in the electroacupuncture treatment group and the drug conventional treatment group.

A total of 4^[7,8,13,14]^ studies reported the outcome index of angina treatment efficacy, and the clinical heterogeneity test was performed for these 4 studies (*I*^2^ = 0%, *P* = .89), and the results of the heterogeneity test suggested that the heterogeneity of the above 4 studies was small, so the fixed-effects model was used to combine the effect sizes, and the results of the Meta-analysis showed that the RR = 1.19, 95% CI = [1.09, 1.31], *P* = .0002 < 0.05, the difference was statistically significant, as shown in the figure, which implies that the intervention group was more effective than the control group in improving clinical symptoms of angina pectoris (Fig. 3).

#### 3.3.2. Electrocardiographic clinical treatment efficiency.

**Figure 4.**
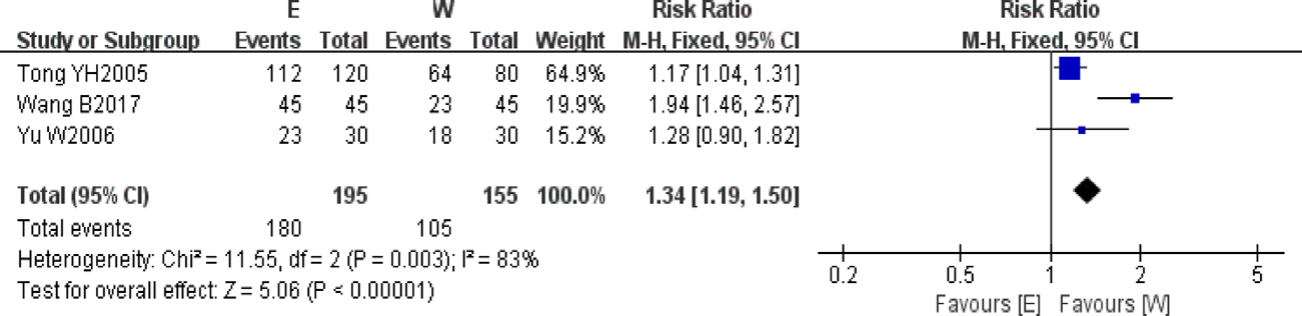
Comparison of electrocardiogram efficacy between electroacupuncture treatment group and western medicine conventional treatment group.

A total of 3^[9,12,14]^ research studies reported the outcome index of ECG treatment efficiency, and high heterogeneity was found in the above 3 studies by heterogeneity test (*I*^2^ = 83%, *P* = .003), so Meta-analysis was performed using random effects model, and the results showed that RR = 1.34, 95% CI = [1.19, 1.50], *P* = .00001, and the difference was statistically significant, suggesting that the intervention group using electroacupuncture combined with western medicine treatment was better than the control group using western medicine treatment alone in terms of improvement of patients’ electrocardiograms (Fig. 4).

#### 3.3.3. Visual analogue score.

**Figure 5.**
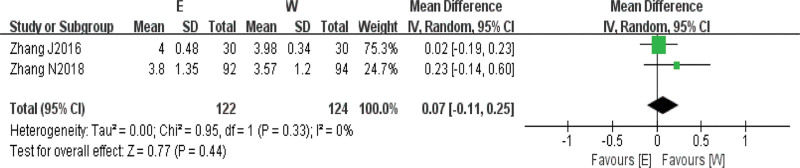
Comparison of VAS scores between electroacupuncture treatment group and western medicine conventional treatment group. VAS = visual analog score.

Two^[10,11]^ studies reported visual analogue scores as an outcome indicator in patients with stable angina, and before Meta-analysis, baseline period agreement tests were performed for both studies, and the results were as follows: there was no heterogeneity in the baseline period effect sizes for VAS scores in the 2 studies (*I*^2^ = 0%, *P* = .33), so the fixed-effects model was chosen to combine effect sizes, as seen in the forest plot of Figure 5, which showed that the intervention group patients had better VAS scores than control patients after treatment, but it was not statistically significant (*Z* = 0.77, *P* = .44 > 0.05), suggesting that although electroacupuncture combined with conventional drug treatment could reduce VAS scores, the reduction did not reach statistical significance, that is, from a statistical point of view, there was no significant difference between electroacupuncture combined with conventional drug treatment compared with conventional drug treatment (Fig. 5).

#### 3.3.4. Seattle Angina Scale score.

**Figure 6.**
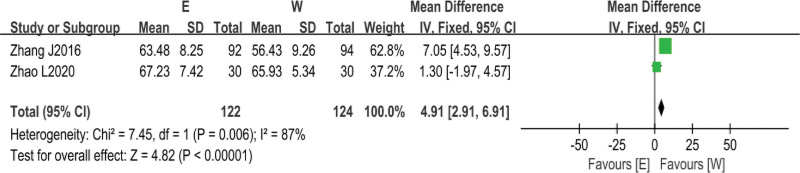
Comparison of SQA scores between electroacupuncture treatment group and western medicine conventional treatment group. SQA = Seattle Angina Scale score.

Two^[9,10]^ studies reported Seattle Angina Scale score (SQA) as an outcome index, and since the results of the baseline period agreement test of the above 2 studies suggested no heterogeneity in the effect size (*I*^2^ = 0%, *P* = .82), Meta-analysis was performed using a fixed-effects model, and the results suggested that the SQA score of patients in the intervention group after electroacupuncture combined with conventional western drug therapy was higher than that of the control group of conventional western drug-treated patients (*Z* = 4.82, *P* < .00001), and the difference was statistically significant, suggesting that electroacupuncture combined with conventional drug treatment could significantly improve patients’ angina symptoms (Fig. 6).

### 3.4. Sensitivity analysis

Sensitivity analysis of the 3^[7,10,13]^ studies using ECG clinical treatment efficiency as an outcome indicator using a single study-by-study exclusion method showed that Meta-analysis heterogeneity decreased when Wang B study was excluded (*I*^2^ = 0%, *P* = .40). Sensitivity analysis using the same method for the 2 studies using the SQA as an outcome indicator revealed no change in meta-analytic heterogeneity.

### 3.5. Publication bias analysis

**Figure 7.**
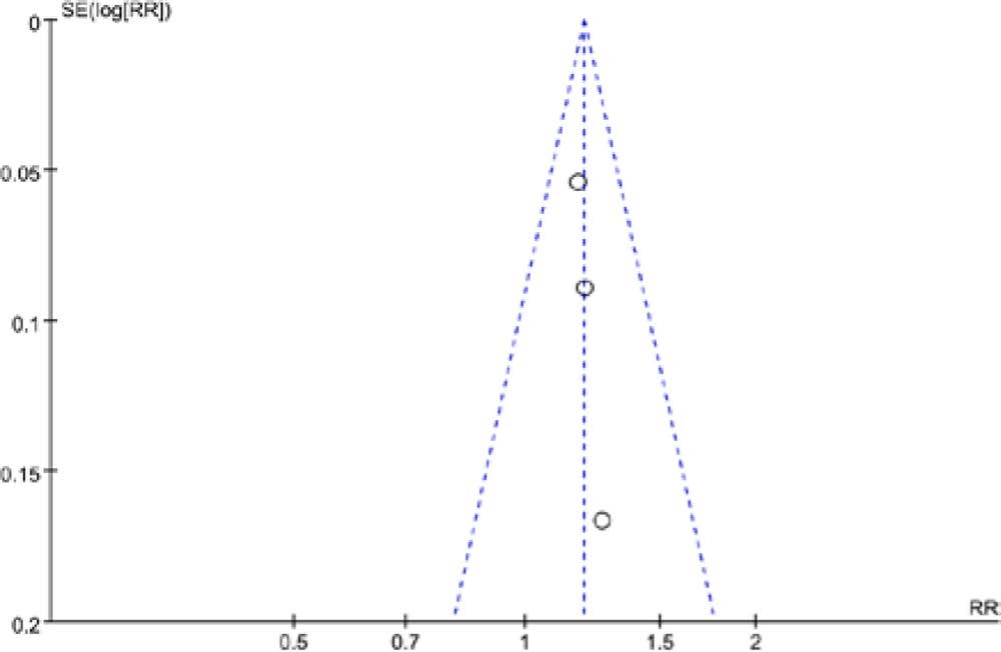
Clinical symptoms of angina pectoris efficacy funnel chart.

**Figure 8.**
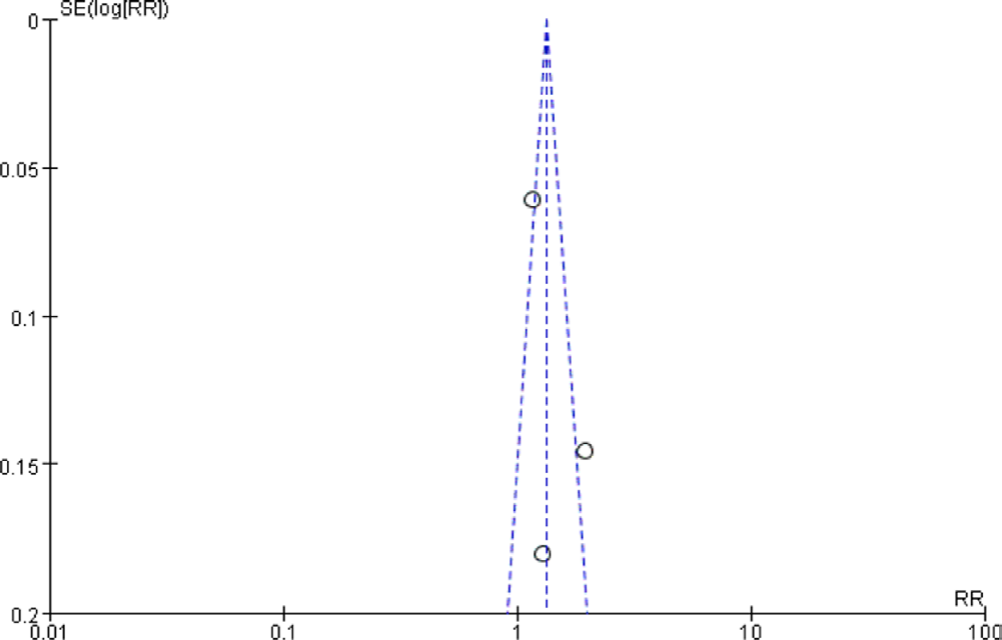
ECG clinical efficacy funnel chart. ECG = electrocardiogram.

**Figure 9.**
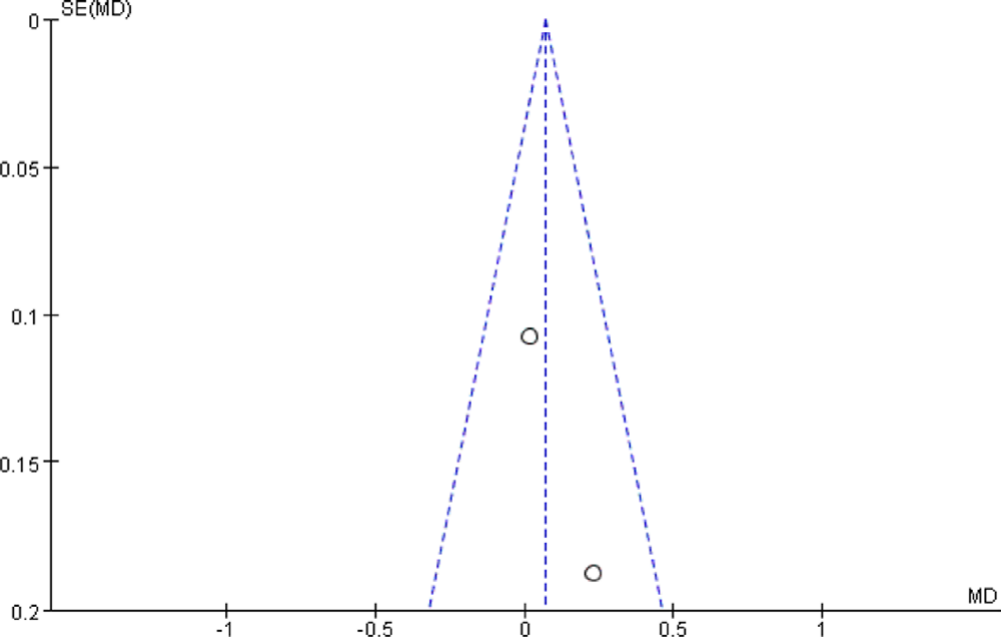
VAS funnel chart. VAS = visual analog score.

**Figure 10.**
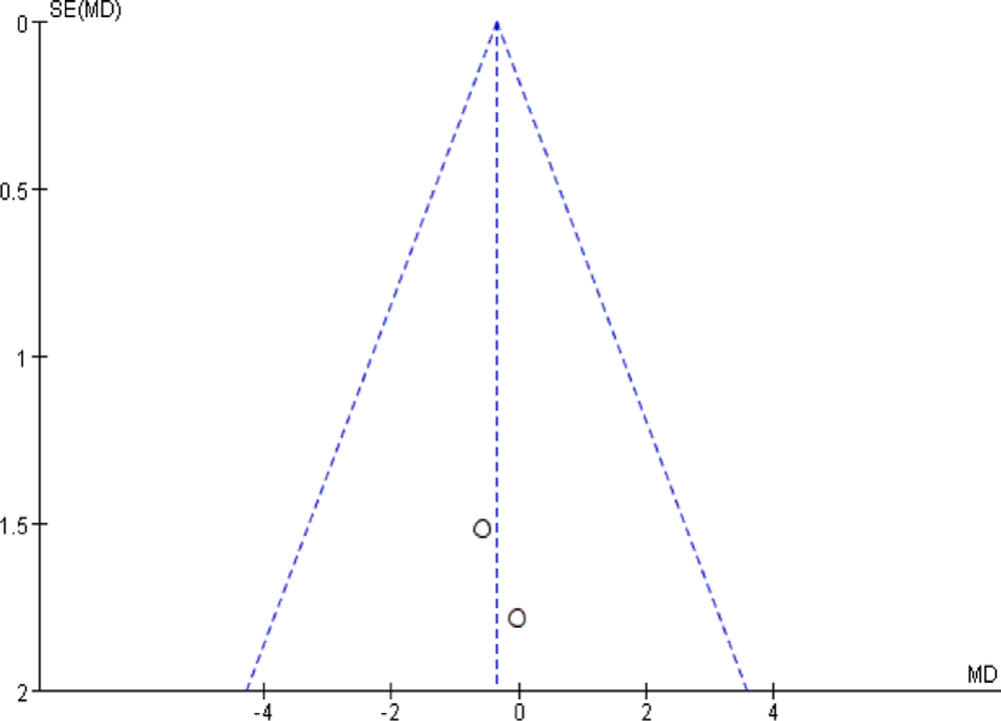
SQA funnel chart. SQA = Seattle Angina Scale score.

The funnel plot was used to analyze the publication bias for improving the clinical symptom efficiency of angina pectoris, the clinical treatment efficiency of electrocardiography, the VAS and the SQA, and the results showed that the left-right distribution of the clinical symptom efficiency of angina pectoris (Fig. 7), the clinical treatment efficiency of electrocardiography (Fig. 8) and the SQS funnel plot were symmetrical (Fig. 9), and the left-right distribution of the VAS funnel plot was asymmetrical (Fig. 10), suggesting the possibility of publication bias.

### 3.6. Related adverse reactions

#### 3.6.1. Adverse drug reactions.

Two^[10,11]^ studies reported adverse drug-related information for stable angina relief. Drug-related adverse reactions were mainly abdominal pain, chest tightness, dyspnea, and heart failure, but there were no serious adverse events.

#### 3.6.2. Electroacupuncture adverse reactions.

One^[9]^ study reported 2 cases of acupuncture-related adverse events during the study. Acupuncture-related adverse events mainly included dizziness and local pain, and no other serious adverse events were observed.

#### 3.6.3. Serious adverse events.

One^[8]^ study reported 1 subject died 9 days after enrollment due to acute myocardial infarction (Table 4).

**Table 4:** Needle stick adverse event report.

Studies	Intervention group	Control group	Sample size	Number of adverse events in the intervention group	Number of adverse events in the control group
Zhang J2016	Electroacupuncture + basic medication	Basic drug therapy: ① Antiplatelet therapy ② Beta-blockers ③ ACEI ④ Lipid-lowering therapy	101	0	1
Zhao L2020	Electroacupuncture + basic medication	Basic drug therapy: ① Antiplatelet therapy ② Beta-blockers ③ ACEI ④ Lipid-lowering therapy	60	2	0
Han LH2020	Electroacupuncture + basic medication	Basic drug therapy: ① Antiplatelet therapy ② Beta-blockers ③ ACEI ④ Lipid-lowering therapy	204	7	19
Wang B2017	Electroacupuncture + basic medication	Basic drug therapy: ① Antiplatelet therapy ② Beta-blockers ③ ACEI ④ Lipid-lowering therapy	90	0	26

ACEI = angiotensin-converting enzyme inhibitors.

## 4. Discussion

Clinical trials and mechanism of action studies at home and abroad have shown that acupuncture treatment improves microcirculation by improving several blood rheological indicators such as fibrinogen, plasma viscosity, red blood cell pressure and aggregation index in patients with angina pectoris,^[15]^ thereby reducing the frequency of angina attacks,^[16]^ decreasing the degree of angina pain, improving clinical symptoms and ECG of angina pectoris,^[16]^ and improving the quality of life of patients with angina pectoris.^[17]^ Electroacupuncture, as a new physical therapy measure,^[18]^ uses pulsed current to unblock the meridians and qi and blood at the acupuncture site on the basis of acupuncture to obtain qi, regulate the function of the internal organs, promote blood circulation, and thus achieve relief of patients’ angina symptoms.^[15]^ The present study was conducted to explore the effectiveness and safety of electroacupuncture in the treatment of angina pectoris in CHD by systematically evaluating previous articles on electroacupuncture in the treatment of angina pectoris in CHD,^[18]^ to find evidence-based medical evidence on electroacupuncture in the treatment of angina pectoris in CHD, and to further provide a basis for clinical and scientific research.^[19]^

A total of 7 randomized controlled trials of electroacupuncture combined with conventional drugs for the treatment of stable angina were included in this study to evaluate 5 outcome indicators of clinical efficiency, ECG clinical treatment efficiency, VAS, SQA, and incidence of adverse effects of electroacupuncture combined with conventional drugs in patients with stable angina. From the current evidence, electroacupuncture combined with conventional drug therapy is significantly more effective than conventional drug therapy alone, especially in improving clinical symptoms of angina pectoris, improving patients’ ECG performance, reducing the frequency of angina pectoris episodes, and reducing the number of angina pectoris episodes, while possessing safety and economic characteristics.

This study found large heterogeneity in some of the outcome indicators, which, upon further investigation, may be due to the following reasons: the study groups included in this study were all from China, the study groups were homogeneous, and there were differences in age, gender, disease duration, sample size, treatment drugs, duration of treatment, and outcome indicators among the populations included in different studies, making the studies not on a uniform baseline, which may have an impact on the analysis results.

The study has certain limitations, mainly in the following areas: the low quality of the literature included in the study, the small sample size, no multicenter, large-sample randomized controlled studies or real-world studies, the inconsistent efficacy indicators used across studies, the lack of blinding and allocation concealment methods in most trials, the treatment of excluded and dislodged cases not clearly reported, and the lack of a rigorous scientific design. In addition, there was a lack of observation and follow-up of long-term patient outcomes in the studies.

## 5. Conclusions

Current research evidence suggests that electroacupuncture combined with conventional drugs can significantly reduce the frequency of angina attacks, decrease the degree of angina pain, improve angina clinical-related symptoms and ECG, and improve the quality of life of patients with angina compared with drug therapy alone. Due to the small number and low quality of literature included in the study, the authenticity and reliability of this study urgently need more standardized design, rigorous multicenter, large-sample randomized controlled trials and real-world studies to verify. It is recommended that future clinical trials on electroacupuncture for stable angina pectoris should use high-quality clinical trial designs and be implemented with reference to international standards as much as possible, such as using the Cochrane Risk of Bias Assessment Tool and Consort to design and evaluate trial protocols and allocation concealment methods, and improving baseline patient data and efficacy evaluation indexes with reference to international standards, in order to reduce various potential risks of bias. to reduce the potential risk of bias and improve the quality of studies related to electroacupuncture for stable angina pectoris.

## Acknowledgments

We thank the authors and patients for their efforts in this study and the editors and reviewers for their patience in reviewing the manuscript.

## Author contributions

**Data curation:** Wenfang Zhong.

**Formal analysis:** Shuwen Pang, Jing Qian.

**Funding acquisition:** Shuwen Pang.

**Investigation:** Shuwen Pang, Yajuan Lv.

**Methodology:** Shuwen Pang, Yajuan Lv, Yueli Zhao, Jiahui Zhong.

**Project administration:** Shuwen Pang.

**Resources:** Shuwen Pang.

**Software:** Shuwen Pang.

**Supervision:** Shuwen Pang, Weiyi Huang.

**Validation:** Shuwen Pang.

**Visualization:** Shuwen Pang.

**Writing – original draft:** Shuwen Pang.

**Writing – review & editing:** Shuwen Pang.
